# Rock River Union Medical Society

**Published:** 1855-08

**Authors:** Oliver Everett, G. W. Phillips

**Affiliations:** Pres’t.; Sec’y.


### MEDICAL NEWS.

*Rock River Union Medical Society.*

The physicians and surgeons of Lee, Whiteside, and Ogle counties, met in convention at Dixon, June 27th, 1855, pursuant to a call previously given.

Present,—Drs. J. B. Nash, G. W. Phillips, N. W. Abbott, Oliver Everett, J. W. Velie, H. Wasson, Dixon; J. A. Jackson, T. M. Crombie, Amboy; A. S. Hudson, Sterling; H C. Donaldson, Como; W. W. Winters, Milligeville; P. K. Guild, Lee Centre.

The convention organized. A constitution was reported, considered and adopted.

Article 1st reads, This association shall be known as the *Rock River Union Medical Society.*

Article 2d reads, All physicians and surgeons of Lee, Whiteside, Ogle, and adjoining counties, if of good, moral, and professional standing, on presentation of their diplomas, from any legally organized Medical College, or County, or State license, from any legalized Medical Board, or satisfactory credentials from such source in other counties. may become members by signing their names to the Constitution ; but no one shall be entitled to membership, whose practice is based upon an exclusive dogma, to the rejection of the accumulated experience of the profession, and of the aids furnished by anatomy, physiology, pathology, and chemistry.

Article 7th of By-Laws reads, The members of this society, in their intercourse with each other, and the public, shall be guided by the code of ethics adopted by the Nationl Medical Association.

The officers elected for the ensuing year are Dr. Oliver Everett, President; Dr. A. S. Hudson, Vice-President; Dr. G. W. Phillips, Recording and Corresponding Secretery; Dr. J. B. Nash, Treasurer; Drs. N. W. Abbott, A. S. Hudson, and J. A. Jackson, Censors.

Drs. O. Everett, G. W. Phillips, and H. Warron, were appointed a committee to prepare a report for the next annual meeting upon the following subject :—What influence has the settlement and cultivation of this country had in modifying the general type of desease.

Drs. N. W. Abbott and J. A. Jackson were appointed, each to prepare a paper upon some medical subject, to be read at the next meeting, which was appointed, second Wednesday of November next, the 14th of the month, at Dixon.

Dr. A. S. Hudson was appointed to deliver the address at the next annual meeting, to be held the second Wednesday of June, 1856, at Dixon.

Dr. N. W. Abbott was appointed as delegate to attend the next annual meeting of the State Medical Society.

Dr. A. S. Hudson was chosen as delegate to the next annual meeting of the National Medical Association.

*Resolved*, That a condensed report of the proceedings of this convention be published in the *North-Western Medical and Surgical Journal.*

On motion, ajourned.

OLIVER EVERETT, M. D., Pres't.

G. W. PHILLIPS, M. D., Sec'y.

